# Development of a battery free, solar powered, and energy aware fixed wing unmanned aerial vehicle

**DOI:** 10.1038/s41598-025-90729-2

**Published:** 2025-02-20

**Authors:** Jackson Liller, Rishabh Goel, Abdul Aziz, Josiah Hester, Phuc Nguyen

**Affiliations:** 1 Department of Computer Science and Engineering, University of Texas at Arlington, Arlington, TX USA; 2 Manning College of Information and Computer Sciences, University of Massachusetts Amherst, Amherst, MA USA; 3 Interactive Computing and Computer Science, Georgia Institute of Technology, Atlanta, GA USA

**Keywords:** Sustainability, Energy harvesting

## Abstract

Unmanned Aerial Vehicles (UAVs) hold immense potential across various fields, including precision agriculture, rescue missions, delivery services, weather monitoring, and many more. Despite this promise, the limited flight duration of the current UAVs stands as a significant obstacle to their broadscale deployment. Attempting to extend flight time by solar panel charging during midflight is not viable due to battery limitations and the eventual need for replacement. This paper details our investigation of a battery-free fixed-wing UAV, built from cost-effective off-the-shelf components, that takes off, remains airborne, and lands safely using only solar energy. In particular, we perform a comprehensive analysis and design space exploration in the contemporary solar harvesting context and provide a detailed accounting of the prototype’s mechanical and electrical capabilities. We also derive the Greedy Energy-Aware Control (GEAC) and Predictive Energy-Aware Control (PEAC) solar control algorithm that overcomes power system brownouts and total-loss-of-thrust events, enabling the prototype to perform maneuvers without a battery. Next, we evaluate the developed prototype in a bench-top setting using artificial light to demonstrate the feasibility of batteryless flight, followed by testing in an outdoor setting using natural light. Finally, we analyze the potential for scaling up the evaluation of batteryless UAVs across multiple locations and report our findings.

## Introduction

UAVs enable many exciting applications in precision agriculture, rescue missions, delivery services, and weather monitoring. However, the current architecture and design of electronic UAVs have multiple significant limitations that hinder their widespread adoption, including limited flight duration, heavy battery weight, high costs and frequent battery replacements, temperature sensitivity^1^, environmental impact, payload limitations, and limited range and coverage, among others.

This research investigates a new solution for solving some of these limitations by eliminating the battery from the UAV’s architecture. Indeed, batteries introduce numerous challenges that hinder the advancement of drones, including (1) *weight*, (2) *limited charge cycles*, (3) *charging infrastructure dependence*, (4) *weak ability to handle high inrush current*, and (5) *negative environmental/ecological impacts*, all leading to shorter flight times and constrained mission space. Batteries add significant weight (65% of total weight^2^) due to heavy chemical compounds like lead, lithium-acid, LiCoO2, LiFePO4, and NiMH^3–7^. They degrade after 200-300 charging cycles due to electrochemical reactions^8,9^, causing electrode material degradation, SEI layer formation, and chemical changes, which reduce capacity. Lithium-ion and lithium-polymer batteries are also vulnerable to heat and mechanical stress, potentially leading to breakage, swelling, or fires^10^. Batteries are not ideal for handling inrush currents from motors, as their limited instantaneous power delivery can lead to voltage drops and damage^11^. This issue is highlighted in literature on batteryless small satellite platforms^12^. Designed for steady, long-term energy supply, batteries are unsuitable for rapid power spikes required by motor-driven machinery^13,14^. Lastly, batteries wear out after 3-5 years, even with careful recharging and storage^15,16^. Replacing and disposing of them is inconvenient, expensive, and environmentally irresponsible.

In this project, we propose to investigate the development of a battery-free UAV that can survive in the air and sustain long-term missions by harvesting solar energy, eliminating the need for battery recharging or replacement. This UAV stores harvested energy in an array of capacitors, which are lightweight, can endure millions of charging cycles, and intelligently regulate the energy for all operations, including sensing, flying, and computing. By harvesting solar energy to fly, battery-free UAVs will enable longer missions like precision agriculture, forest wildlife censusing, and search and rescue, among many others. For example, in precision agriculture, commercial farms in the US operate upon many thousands of acres of crop fields, and with the advent of IoT sensors it has become common for such farms to deploy constellations of sensing equipment to monitor their crops. Depending on the constellations, it may be necessary to reside in close proximity to connect to the sensors and download their data payloads; however, with larger facilities, this can become a burden, preventing practical deployment of these constellations. Battery-free UAVs could sustain when the sun is up, visiting each critical constellation node and loitering as long as necessary to download the data payload without concern for critical battery stores. This would also be true of other applications; monitoring air quality in cities could be done with a single drone outfitted with the right sensors, and wildfires could be tracked in real-time by a drone with global positioning systems (GPS) and infrared cameras.

However, building a battery-free UAV faces multiple technical challenges going beyond massive engineering efforts:

*C1. Enormous design space.* Designing, implementing, debugging, and evaluating the preliminary system from scratch revealed many pain points and obstacles in making battery-free UAV designs robust. Imperfections in materials, inaccuracies in motor outputs, wing sizes and shapes, energy storage sizes, and payload were all challenging to balance to even get the prototype airborne for a few seconds.

*C2. Overall knowledge of battery-free drones is limited.* There have been efforts to harvest energy from the environment to charge UAVs while flying, but battery-free design and implementation of UAVs are limited. To the best of our knowledge, the flapping wing battery-free UAV^17^ is the closest and only related work, but this UAV design is fragile and does not support sufficient load for real-world applications.

*C3. Energy must be sought out.* The impact of the sun’s angle and environmental factors on harvested energy is poorly understood. With appropriate controllers, energy-seeking behavior must be implemented to enable long flight times. In order for the UAV to be reliably airborne, a sophisticated technique that takes measures from the UAV’s body signals (e.g., wing’s flexibility, flying angle, frequency), and environmental impact (e.g, sun angle, wind, rain, cloud) to control the UAV’s flying and navigating behaviors.

This paper addresses the above challenges and makes the following contributions:

- We study, design, and fabricate the first battery-free fixed-wing UAV that is powered completely by harvested energy to perform its sensing, computing, and flying tasks without a battery.
- We develop a power system based on contemporary solar and supercapacitor technology to control and regulate the power efficiently to deliver energy across multiple components of the complex UAV architecture
- We develop two novel energy-aware control algorithms that enables the UAV to operate reliably with intermittent power. This is a challenging task because existing intermittent computing techniques cannot be directly applied to the high voltage, inrush current, and precision demands required by the proposed aerodynamic system.
- We evaluate the developed prototype using in-lab and outdoor testing to confirm the UAV’s ability to take off, fly, and land safely without a battery, and use historical solar weather records to analyze the UAV’s deployability across the world.

*Potential applications*. Battery-free UAVs have the potential for long flight times, low/no maintenance, reduced costs, and even environmental friendliness. Batteries are a major hindrance to flight endurance^18^. Due to battery degradation, frequent whole battery replacements are expensive financially and ecologically. Numerous large-scale drone applications are emerging or proposed, such as automated delivery^19–24^, large scale agriculture, and wildfire, urban spaces, and environment monitoring^25–28^. Each of these emerging applications requires enormous fleets of small craft drones, potentially tens of millions of small craft— ballooning battery waste, adding high maintenance costs, requiring frequent battery replacements, and sophisticated charging infrastructure for longer flights, which are not friendly to humans, financing, or the environment.

## Results

The results presented in this section showcase advancements that push the boundaries of battery-free UAV technology. Unlike previous studies that rely on batteries for energy storage, this work demonstrates a fully operational UAV powered solely by harvested solar energy and supported by innovative control algorithms designed to mitigate power interruptions. Specifically, the results highlight the development and implementation of two novel energy-aware control strategies, which address challenges of power intermittency by preventing brownouts and minimizing total-loss-of-thrust events. Table 1 highlights the records that were set by the batteryless UAV under each control schema used. This section is organized to provide a comprehensive evaluation of the proposed battery-free UAV. It begins with an aerodynamic assessment, followed by an analysis of the power system’s performance and the effectiveness of the energy-aware control algorithms. Finally, a scalability analysis is presented to explore the potential for deploying the UAV in various real-world environments.

**Table 1:** Batteryless UAV features and records by control algorithm.

Feature/throttle control	Naive	Greedy energy-aware control	Predictive energy-aware control
Brownout mitigation	$\textsf {x}$	$\checkmark$	$\checkmark$
Total-loss-of-thrust mitigation	$\textsf {x}$	$\textsf {x}$	$\checkmark$
Best ground speed (m/s)	10	10	15
Best air speed (m/s)	-	-	15

**Fig. 1:**
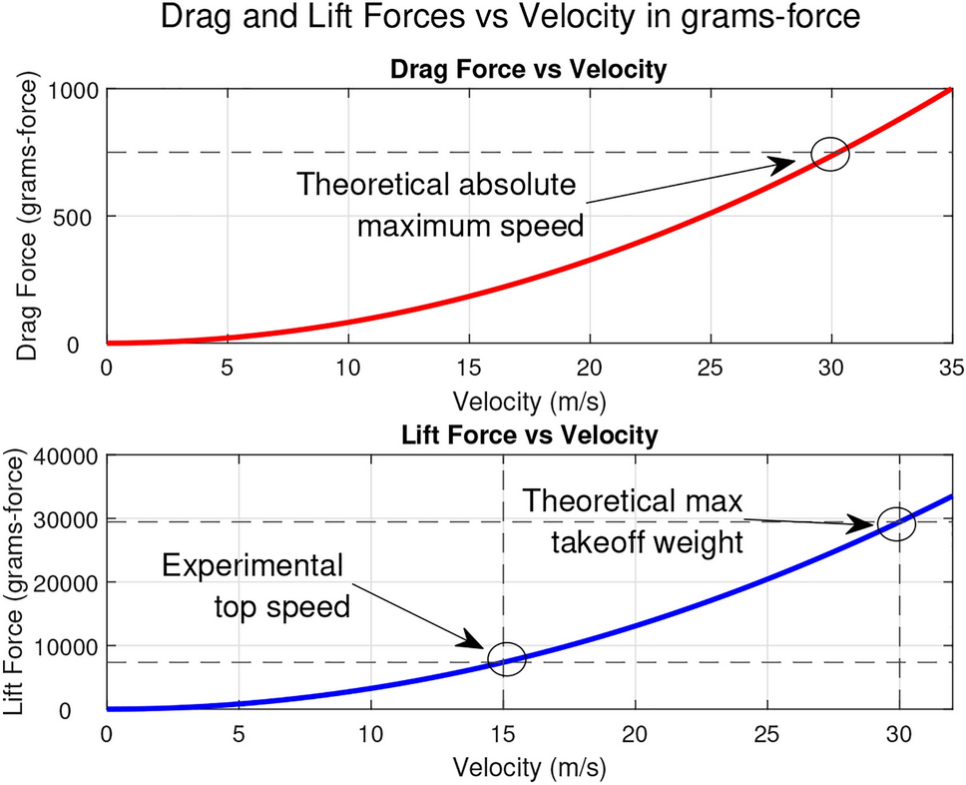
Graphical derivation of maximum velocities and lift forces.

**Fig. 2:**
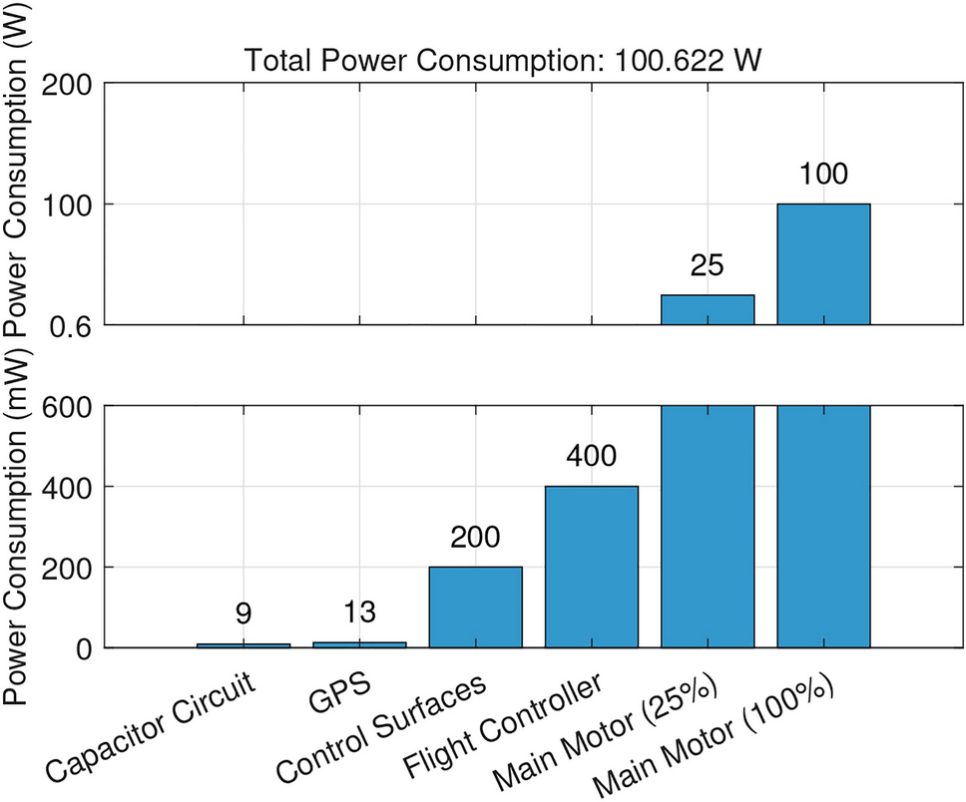
Power distribution.

**Fig. 3:**
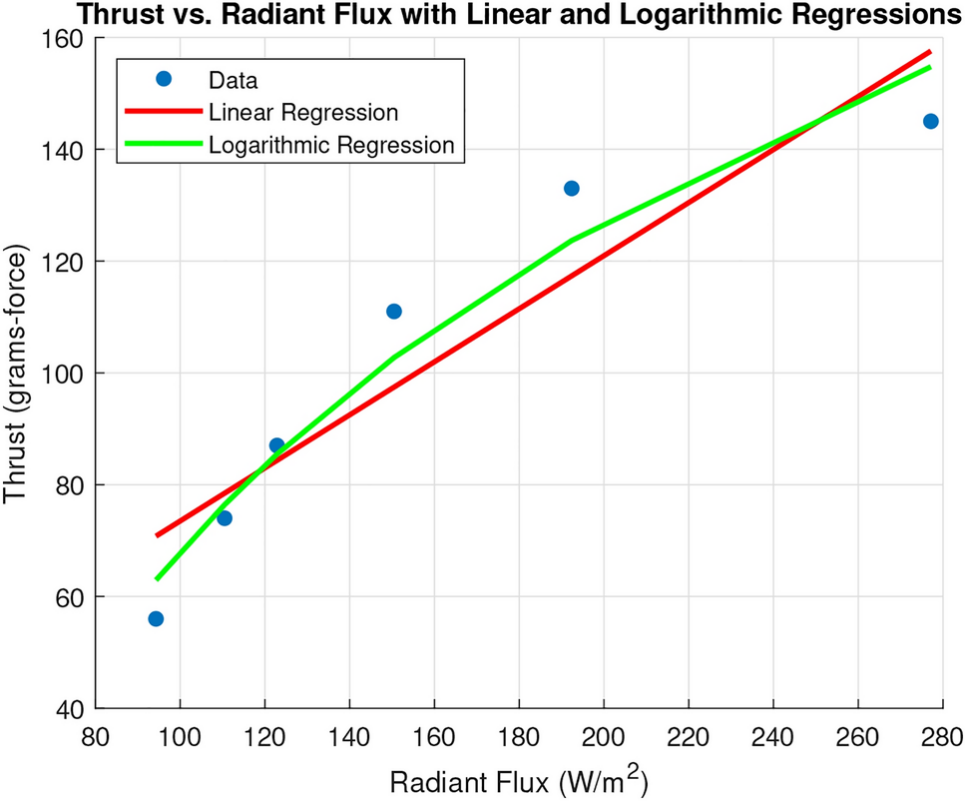
Radiant flux vs. thurst.

### Aerodynamic evaluation

Test flights produced a best altitude of approximately 3 meters and a top speed of 15 m/s. Figure 1 highlights the disparity between this experimental top speed and the theoretical top speed discussed in Section 3.4.1. At this speed, we see a considerably smaller takeoff weight:

1 $$\begin{aligned} L_{\text {max, experimental}} = \frac{1}{2} \rho V^2 S C_L \approx 7341 \text { grams-force} \end{aligned}$$

The deficit between the theoretical top speed (described in further detail in Section 3.4.1) and $L_{max,experimental}$ arises from some simplifying assumptions made in the derivation. Although the direct effects of body drag cannot be quantified without specialized testing equipment, it is clear that some unaccounted-for drag is acting on the drone; however, the assumption of constant $T_{max}$ is just as suspect. Section 3.3 examines this assumption in more detail.

### Power system evaluation

#### Power generation

 Figure 2 shows the power distribution of all components on the drone. Obviously, the main motor consumes the most power and changes its requirements throughout its throttle curve. The most important power system relationship for the batteryless UAV is how effectively it can convert radiant flux into thrust. To determine this relationship, a series of experiments were conducted using the outlined experimental setup in which the height of the halogen lamps was adjusted to simulate varying radiant flux. It is worth noting that the last data point was taken from a solar noon reading outdoors instead of from the halogen lamp setup. After a long period of charging the capacitor bank, the main motor was set to max throttle with GEAC modulating throttle control such that the capacitor voltage could not drop below 10.5 V. After a short burst of high throttle, GEAC would force the motor to seek the throttle setting which drew only the power that could be instantaneously produced by the solar panels given the radiant flux available. The corresponding thrust produced by this throttle setting was recorded by the load cell and a lux measurement was taken and converted to radiant flux according to the formula: $\Phi _e = \frac{E_v}{K}$, where $\Phi _e$ is the radiant flux in watts per square meter ($\mathrm{W/m}^2$), $E_v$ is the illuminance in lux (lx), *K* is the luminous efficacy in lumens per watt (lm/W), which depends on the light source. The luminous efficacy of the halogen lamps was estimated to be equivalent to that of the sun, 105 lm/W. Conventionally, the relationship between radiant flux and solar panel output is linear; however, the relationship between power input and thrust on the motor is logarithmic. Both regressions are shown in Fig. 3. This data suggests that in favorable conditions, the batteryless UAV ought to be capable of producing 350 grams-force of thrust indefinitely.

**Fig. 4:**
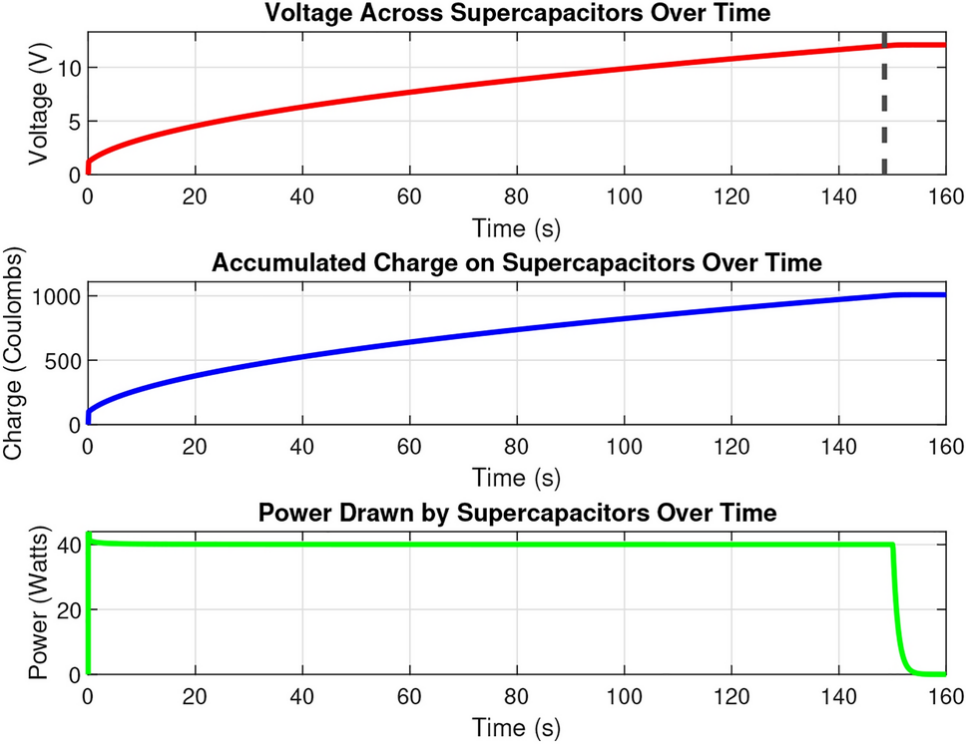
Supercapacitor charging characteristics at best experimental solar harvesting levels.

**Fig. 5:**
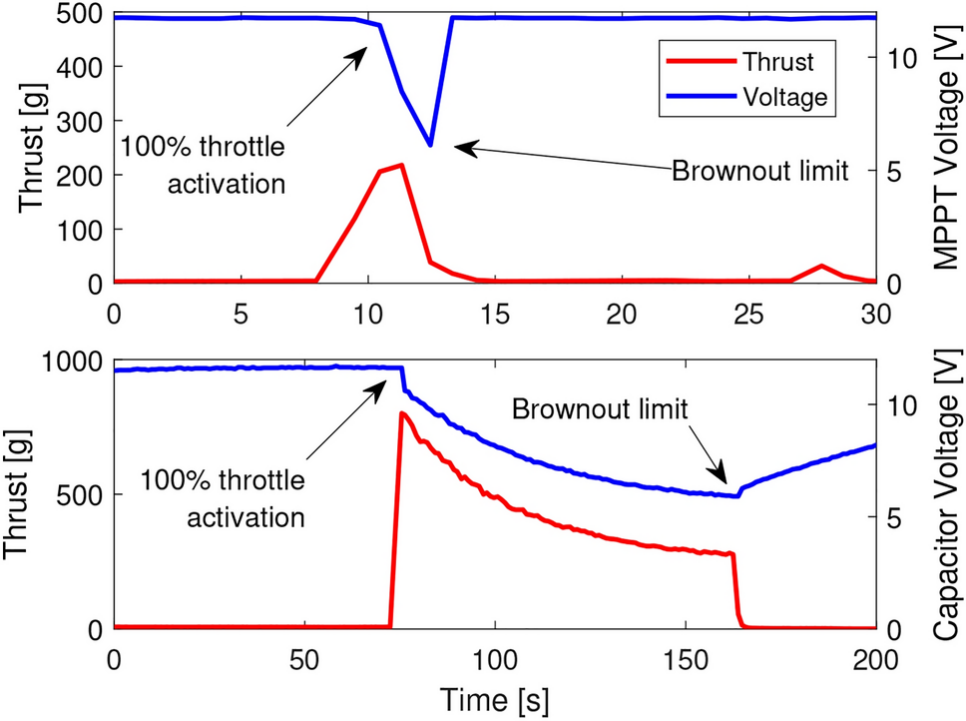
Solar harvesting without capacitors (top) and with super-capacitors (bottom).

**Fig. 6:**
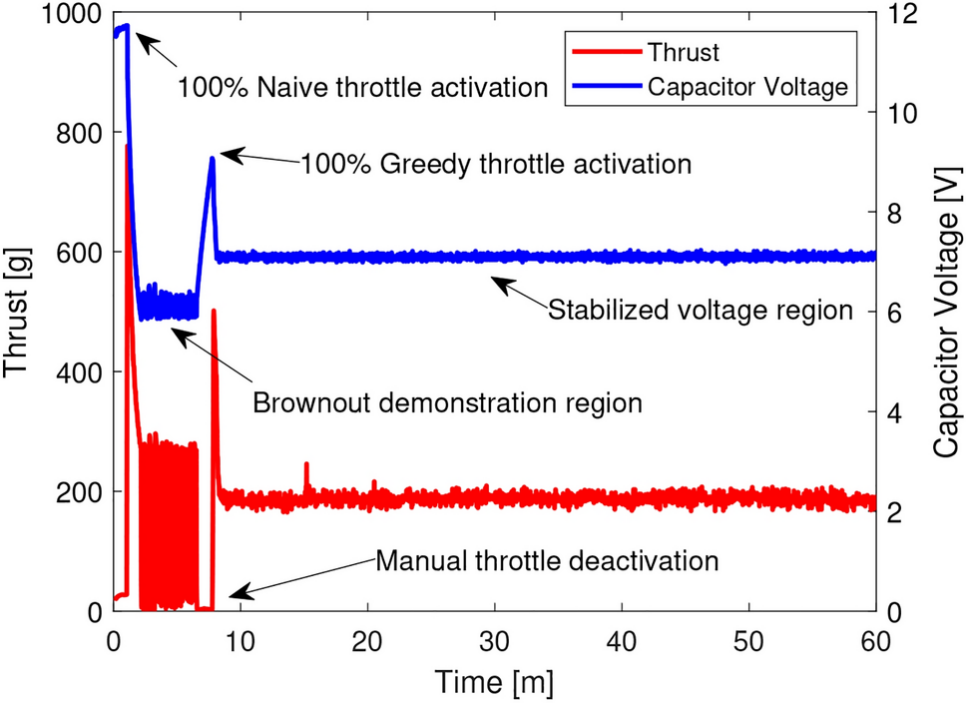
Brown-out prevention using Greedy-EAC algorithm.

**Fig. 7:**
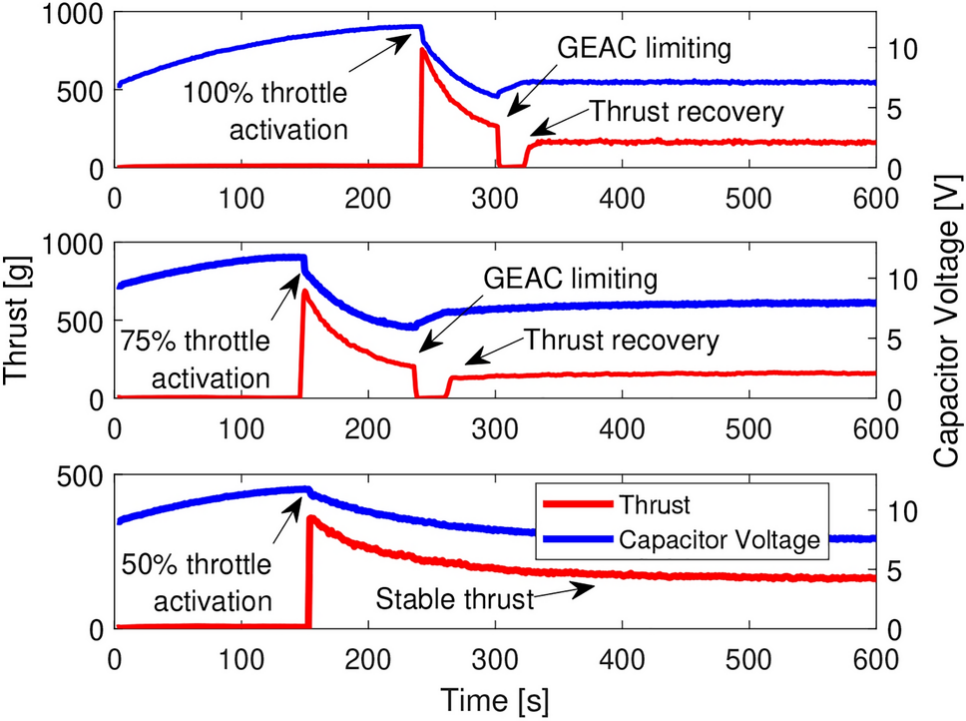
Greedy-EAC at 100% throttle (top), 75% throttle (middle), and 50% throttle (bottom).

**Fig. 8:**
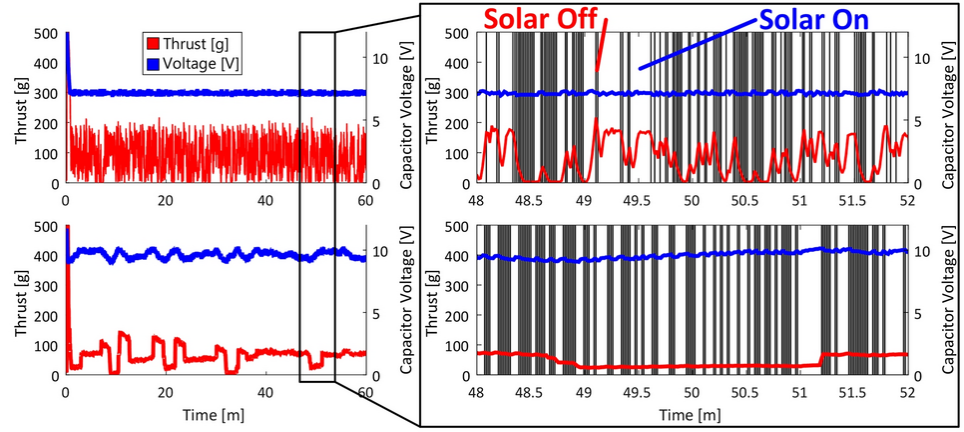
Total loss of thrust prevention demonstration of Greedy-EAC (top) and Predictive-EAC (bottom) with lamp state visualization (right).

#### Power storage

 Figure 4 summarizes the charging characteristics of the supercapacitor. The supercapacitor bank requires time before takeoff to charge because the supercapacitor voltage is tied to the flight controller voltage. The time it takes to charge is tied to the radiant flux, but the best available power developed by the MPPT during outdoor experiments was 40 W. Using this number, the super capacitors can be charged to the operating voltage of 12 V in 150 seconds. The batteryless UAV requires a super capacitor bank to buffer charge to overcome intermittent events, and to alleviate brownouts caused by the massive inrush current of the motor on powerup. Figure 5 demonstrates the brownout protection and increased power capacity offered by the proposed super capacitor bank. Although the super capacitor bank cannot prevent brownout events entirely on its own, this issue is solved with control algorithms later. Further, it can be seen that nearly four times as much thrust can be developed from solar harvesting with a super capacitor bank as can be without one.

### Control algorithm evaluation

#### Greedy-EAC algorithm

 The main motor of the UAV is capable of drawing enough current to temporarily rob the flight controller of its required operating voltage, causing a “brownout.” Greedy energy aware control is meant to solve the brownout problem for the batteryless UAV, and is demonstrated in Fig. 6. Using the experimental setup outlined in Section 3, the UAV’s throttle was set to 100% without GEAC engagement to demonstrate the tendancy of the motor to brownout the power circuit at high throttle; the motor would draw too much power, the flight controller would reset when the input voltage was too low, the voltage would recover enough to engage the motor again and the cycle would repeat. After five minutes of brownout demonstration, GEAC was engaged with a low voltage threshold of 6 V and a high voltage threshold of 9 V. Once the capacitor bank recovered to 9 V, the motor was allowed to throttle up until the low threshold of 6V was achieved after which GEAC would limit the throttle to prevent further brownouts. This demonstration was allowed to continue to the hour mark to demonstrate the robust ability of GEAC to prevent brownouts.

GEAC demonstrated that preventing brownouts can cause total-loss-of-thrust during intermittent power events, meaning the main motor is powered completely down to prevent an impending brownout. It is better to have less thrust over long periods of time than to have several high bursts of thrust over short periods of time because starting a fully stopped motor uses more power than increasing the rotations per minute (RPM) of a slowly spinning motor. It was theorized that there must be some throttle setting which would only consume the excess instantaneously harvested solar energy, so an experiment was conceived to determine if this behavior could be observed.

During the experiment, the capacitor banks were allowed to charge completely before some level of throttle activation was performed under GEAC supervision. Figure 7 summarizes the results of this experiment, demonstrating that the power system was critically sensitive to the instantaneous change in voltage over time. As the experiment proceeds, the thrust output trends downward since the system voltage also trends downward; however, once the lower limit of the GEAC algorithm is reached the power to the main motor is lowered so fast as to appear to be instantly cut off. Afterward, once the system voltage begins to recover, the main motor is allowed to throttle back up to a stable thrust. Given that higher throttle settings incur higher instantaneous voltage drops, several throttle settings were attempted until no loss of thrust was observed. We can see that 50% throttle activation did not incur a complete loss of thrust for GEAC, suggesting that loss of thrust events could be, if not eliminated, at least minimized.

**Fig. 9:**
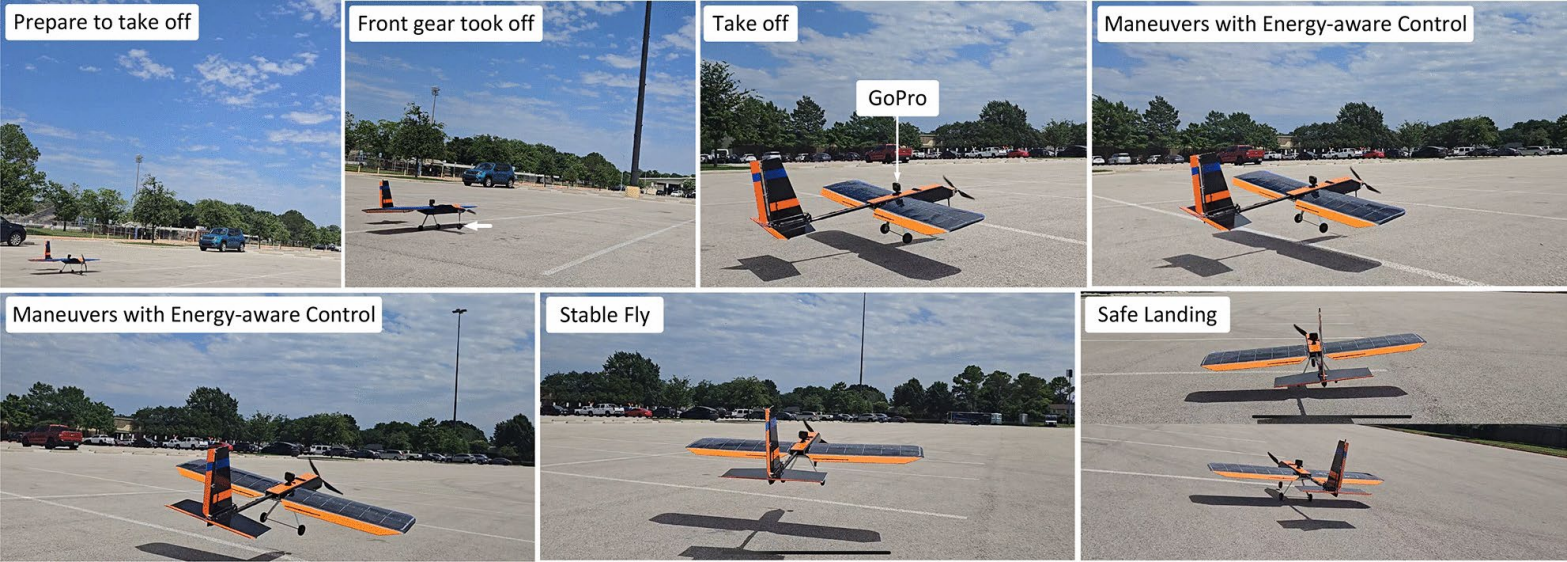
Battery-free UAV performs a complete cycle: run way, take off, fly, and landing.

**Fig. 10:**
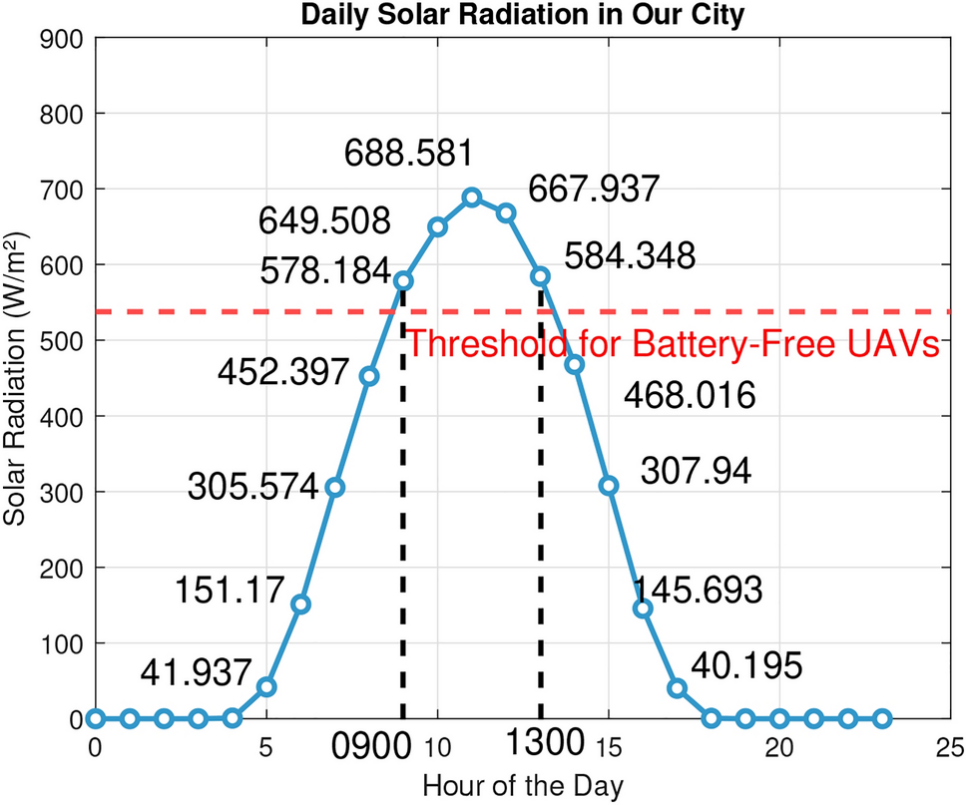
Year-average hourly solar irradiance in Dallas, TX in 2022.

**Fig. 11:**
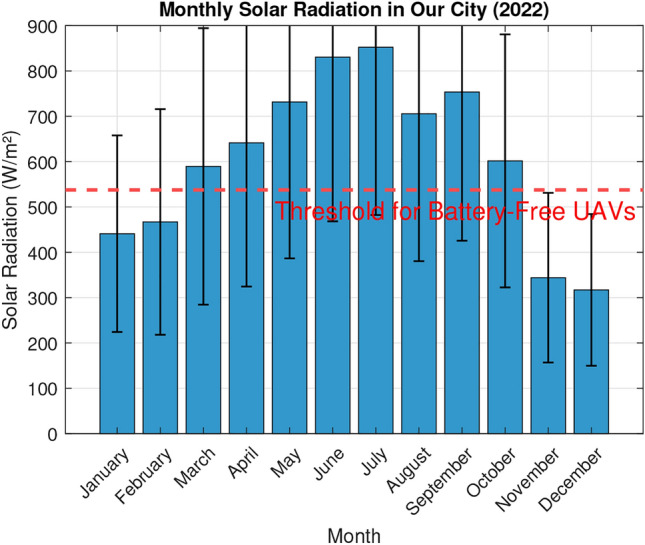
Average monthly solar irradiance (9AM-1PM) in Dallas, TX in 2022.

**Fig. 12:**
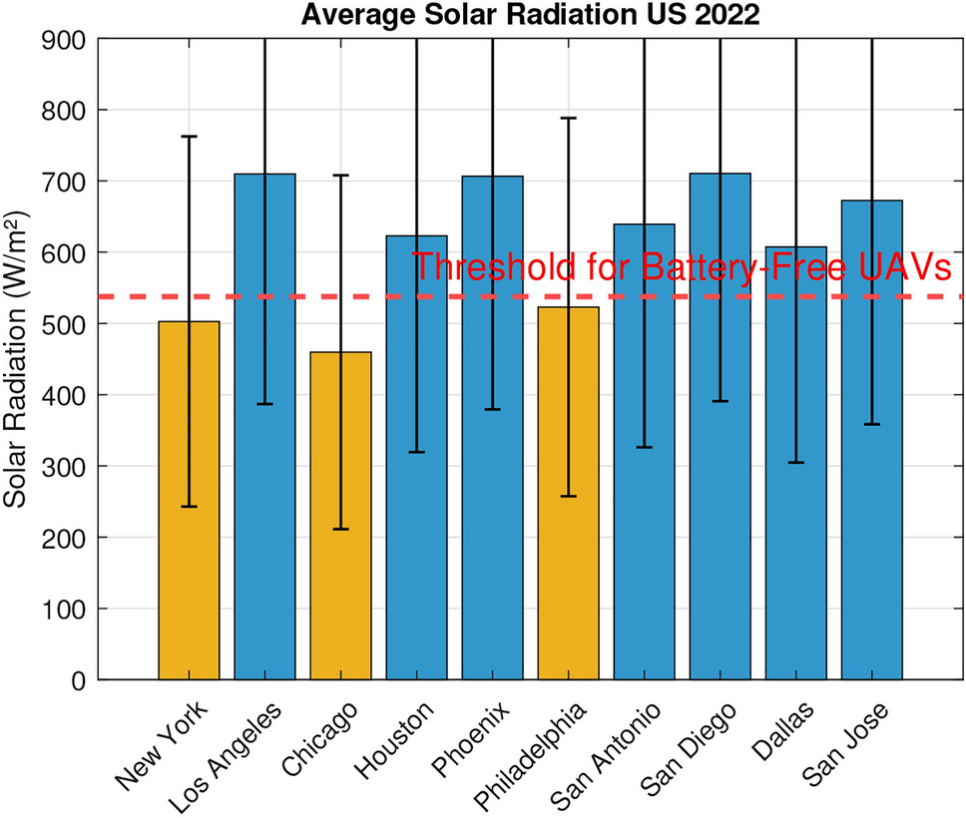
Average solar irradiance (9AM-1PM) in US cities in 2022.

#### Predictive-EAC vs Greedy-EAC algorithm

 PEAC is the natural successor to GEAC, incorporating a software solution for estimating the instantaneous voltage drop and responding to this metric in real-time. PEAC adjusts the throttle to keep the capacitor voltage between two threshold levels, but in addition the algorithm senses the change in voltage by comparing the current voltage reading to the preceding reading. If this change is positive with respect to time, the throttle is allowed to increase, and vice versa. In this way, PEAC minimizes total loss of thrust events by selectively adjusting the throttle setting according to instantaneous power events. An experiment to demonstrate the superiority of PEAC over GEAC in dealing with intermittent power events was devised and is summarized in Fig. 8. The experiment used the experimental setup outlined in Section 3, however the power to the halogen lamps was controlled using a logical relay switch and programmed to randomly switch state, simulating intermittent power events. The zoomed-in view of Fig. 8 includes a visualization of the lamp state during the experiment; intuitively, the bright sections represent a powered lamp state and vice versa. During the experiment, GEAC floated at the low voltage threshold for the entire experiment, greedily consuming any spare power and converting it into short bursts of high thrust and experiencing hundreds of total loss of thrust events. PEAC, however, smoothed the thrust response by slowly adjusting to the lamp states in real-time, totally losing thrust only a handful of times. With the implemented algorithm, the battery-free UAV performs a complete cycle: run way, take off, flight, and landing as illustrated in Fig. 9.

### Scalability analysis

The National Solar Radiation Database (NSRDB) provides high-resolution solar radiation data, including Global Horizontal Irradiance (GHI), Direct Normal Irradiance (DNI), and Diffuse Horizontal Irradiance (DHI). Drawing from multiple satellite and environmental monitoring systems from sources including the National Oceanic and Atmospheric Administration (NOAA) and the National Aeronautics and Space Administration (NASA), the NSRDB offers accurate regional solar radiation information. Our analysis focuses on GHI, a comprehensive measure of total solar energy available on a horizontal surface. Using the Typical GHI Year (TGY) dataset from NSRDB, which represents a typical year based on GHI data, we first collected the GHI values for Our City on an hourly basis over 2022 and averaged by hour, shown in Fig. 10.

**Fig. 13:**
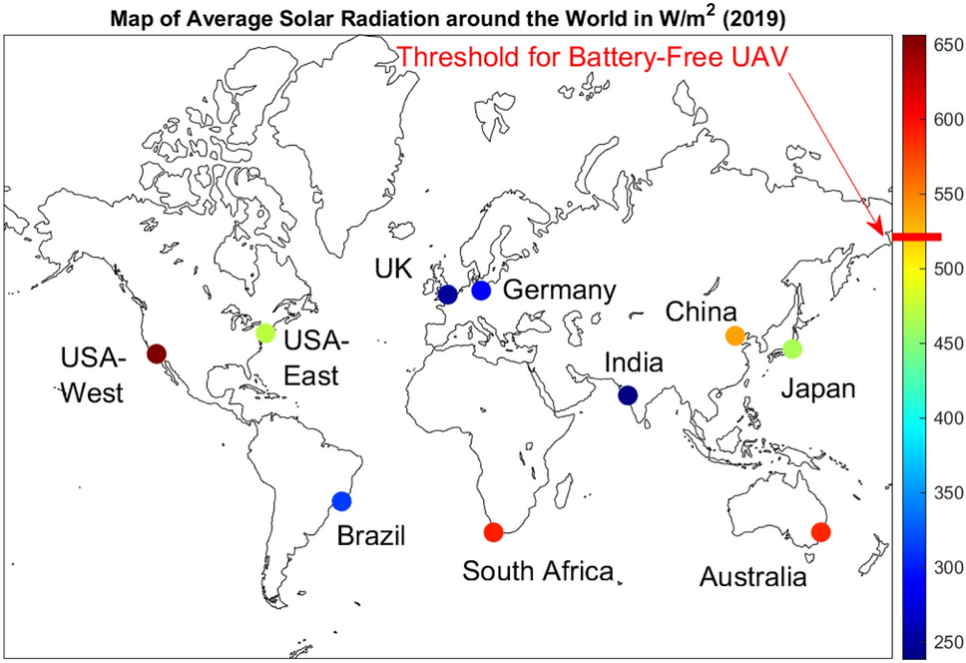
Average solar irradiance for 9AM-1PM around the world in 2019. Map is generated using Worldmap library^29^ in MATLAB 2023b 64-bit Update 7 (23.2.0.2515942).

The threshold for battery-free UAVs was calculated by interpolating the irradiance required to meet the experimental top speed according to the most conservative regression of Fig. 3. To achieve a velocity of 15 m/s, 200 grams-force of thrust is required. Using the linear regression to estimate, the required radiant flux is 537.57 W/$m^2$ This revealed that the best times to fly our prototype were between 0900 hours and 1300 hours. This timeframe was used to estimate the viability of the drone across multiple domains. Because solar irradiance varies widely throughout the year, the threshold was compared to the average irradiance within 0900 to 1300 hours for each month in Our City for the year 2022. Figure 11 graphically represents the results, showing that the UAV would fly on average between March and October, but with the variance in irradiance could fly some days nearly any month but December. A similar analysis was performed for prominent US cities shown in Fig. 12; any bar shown in blue would be likely to experience sufficient irradiance for the UAV to fly, but with variance the drone could fly in any of these cities some days. Next, we collected the GHI values for major global cities using the most recent dataset available (2019)^30^. The data was used to calculate average irradiance values between 0900 and 1300 hours for each country, enabling us to create a world map of average annual solar radiation (Fig. 13). These observations are useful in understanding the possibility and limitations of the current prototype.

## Methods

This section details the methodologies employed in developing and evaluating the proposed battery-free UAV system. To contextualize the work, we first review related efforts that informed the design and implementation of our approach, highlighting the gaps this study aims to address. Following this, we describe the proposed approach, which integrates novel energy-aware algorithms and contemporary solar harvesting techniques into a fixed-wing UAV architecture. A comprehensive system overview is then provided, outlining the design principles and components of the UAV. Finally, we present the experimental setup used to validate the system’s performance, offering insights into how the results in the previous section were obtained.

### Related works

*Wireless charging* One way to make UAV solutions more sustainable is to charge them wirelessly. Wireless power transmission has been in use by the military for nearly a century^31–36^, but it is generally regarded as a novelty due to the extreme inefficiency of the practice. Using an electrical source to generate high power emissions (lasers, microwaves, etc.) can have an efficiency loss of 15-50% and using a state-of-the-art photoelectric converter to transduce those emissions into electrical energy can have efficiency losses of 50% or more. Still, if the incentive is high enough and the source grid is strong enough then the technology can be adapted to the purpose. Researchers have prototyped a multirotor drone and the corresponding ground transmission laser which charges the drone in-flight^37^. The commercial enterprise Global Energy Transmission has created waystations which can be deployed along flight routes to charge drones in-flight^38^, but there are many engineering and logistical challenges to overcome before this method can be adapted for mainstream UAVs. Primarily, since wirelessly transmitted power is highly directional systems to track the UAV and calculate targeting solutions for the emission beam would be required^39,40^, and once these solutions were developed drones would be limited to operational areas inside the effective area of these emission stations.

*Solar-powered UAVs* Solar panels seem like the obvious choice for extending flight times in UAVs, and some projects have already tried applying the batteryless model to other airframes^17^. Modern solar cells are capable of generating power in excess of 200 W/m2^41^, and fixed-wing drones have plenty of surface area for solar cell application thanks to their broadly shaped wings. Furthermore, fixed-wing drones make use of aerodynamic advantages that produce more lift than the thrust developed by the main motors, limiting the need for power hungry motors. Many projects have applied solar harvesting to drones to extend the flight times considerably^18,42,43^, but even after solving the engineering challenges associated with in-flight solar energy harvesting, indefinite flight of the UAV will still be limited by the battery (Table 2). Modern lithium-ion batteries can only recharge around 1000 times before they must be replaced and are highly susceptible to environmental fluctuations in temperature and humidity. Furthermore, batteries can represent as much as 65% of the total weight of a drone, limiting payloads and deployment packages for sensing.

*Harvest energy from powerlines* Typical electrical lines transmit alternating-current (AC) power, oscillating polarity 60 times per second, which induces magnetic fields along their length. Researchers have demonstrated an autonomous drone with an inductive charging harness that attaches to powerlines in flight, using these magnetic fields to charge like inductive smartphone chargers^44^. Their two-hour deployment showed the drone performing missions and charging autonomously from various powerlines. However, the system has a small charge/discharge ratio of modern batteries; it takes longer to charge a lithium-ion battery than it takes to discharge, and this ratio can vary dramatically depending on the electrical load demanded from the battery and the available power to charge the battery.

**Table 2:** A comparison of state-of-the-art related works.

Related work/feature	Battery	Infrastructure reliance	In-flight charging	Airframe	Flight time
NPU Drone^37^	$\checkmark$	$\checkmark$	Laser-induction	Multi-rotor	Indefinite
Hoang et. al.^44^	$\checkmark$	$\checkmark$	Powerline-induction	Multi-rotor	Indefinite
Morton et. al.^43^	$\checkmark$	$\textsf {x}$	Solar	Fixed-wing	Multi-Day
Batteryless Bird^17^	$\textsf { x}$	$\textsf {x}$	Solar	Ornithopter	-

*The limiting factor* Almost all of the aforementioned related works suffer from one common factor: *batteries*. Using batteries to store charge is as natural as putting gasoline in a car’s fuel tank, but as a solution for extending UAV flight times batteries simply fall short. First, batteries can comprise as much as 65% of the weight of typical UAVs^45^ as discussed, increasing the power needs of the lift systems. Second, as flight times become longer and longer thanks to constant recharging efforts the cost of replacement batteries required due to limited lifetime charge cycles will grow. Third, the electrochemical reaction that produces electricity inside of batteries is highly dependent on temperature, limiting the operational ceiling of UAVs due to atmospheric cooling or requiring dedicated temperature maintenance systems on the drone. And finally, once these batteries have lived their useful lives, they become harmful waste that is burdensome to dispose of properly. The solution to this issue is simple: remove the battery entirely. A purely solar-powered UAV offers a compelling solution to address the limitations of battery-powered UAVs. Solar UAVs would integrate solar panels into their wings and contain the hardware to generate electricity efficiently and continuously from sunlight, significantly extending their endurance and operational range by forgoing the cumbersome battery in favor of a capacitor array to buffer charge. This UAV would be free from operational constraints that limit mission areas to locations with infrastructure like power lines and emission stations; anywhere the sun was shining, this drone could fly.

The practical limitations of UAVs must be addressed before they can be employed across different domains as discussed in literature^46–54^. Multiple surveys have been conducted to outline the opportunities and challenges of drone applications, primarily focusing on the use of sensors and machine learning to enable UAVs to efficiently adapt to dynamic environments^55–62^. However, these tasks are computationally intensive and power-hungry, rapidly depleting UAV batteries and severely limiting flight time and endurance. Simply acting as a data relay is also impractical because maintaining the communication channels consumes the limited power available for flights. As a result, UAVs face a trade-off between power allocation for sensing, communication, computation, and flight, resulting in short ranges and frequent recharging or battery swapping.

Alternative power solutions present unique challenges of their own^63–65^. Solar-powered drones can potentially fly indefinitely with sufficient sunlight^66–70^, eliminating the need for ground-based transmitters. Despite the technology’s origins in the 1970s^71^, modern solar-powered UAVs still face challenges such as complexity, cost, and dependency on specific environmental conditions^42,72–75^. Recent advancements, like those by Oettershagen et al.^76^, show the feasibility of solar energy harvesting for extended UAV flights using thin-film photovoltaic panels. However, the low efficiency of these panels^77^ necessitates a long wingspan (5.69m), increasing size, weight, and cost. Additionally, reliance on specific conditions, such as thermal updrafts, restricts general use, and batteries still require eventual replacement. Increasing battery capacity is also not feasible due to the added weight, which reduces flight time as more thrust and power are needed^78^. The concept of batteryless UAVs introduces additional considerations, particularly in managing intermittent power delivery unlike traditional battery-operated systems. Wirelessly-powered UAVs face challenges with range and fluctuations, while solar-powered UAVs contend with varying sunlight intensity. These interruptions can cause power gaps that the UAV must manage without failing and crashing. Rotary-wing UAVs lose lift immediately without power, while fixed-wing drones can glide, making them better suited for batteryless operations with improved flight endurance. However, designing batteryless UAVs, especially smaller ones, presents unique challenges including Reynolds-number sensitivity^79^ and structural design considerations^60–62^.

### Background and motivations

*Why fixed-wing?* Fixed-wing UAVs are ideal for battery-free design due to their inherent design advantages that maximize energy efficiency and flight performance^80^ and the limitation of existing solar harvesting technology. Fixed-wing UAVs have a high lift-to-drag ratio and can glide using natural air currents, reducing the need for continuous power input. This is important for battery-free systems that rely on intermittent power sources like solar panels. The ample surface area of fixed-wing UAVs allows for effective solar energy harvesting without impeding flight, and their consistent forward motion ensures optimal sunlight exposure. Additionally, fixed-wing UAVs can utilize wind and thermal updrafts to gain altitude without consuming extra energy. They generally have longer flight times due to their efficient energy use and ability to glide and soar. Furthermore, fixed-wing UAVs can carry larger payloads, making them a more prefferable platform.

**Fig. 14:**
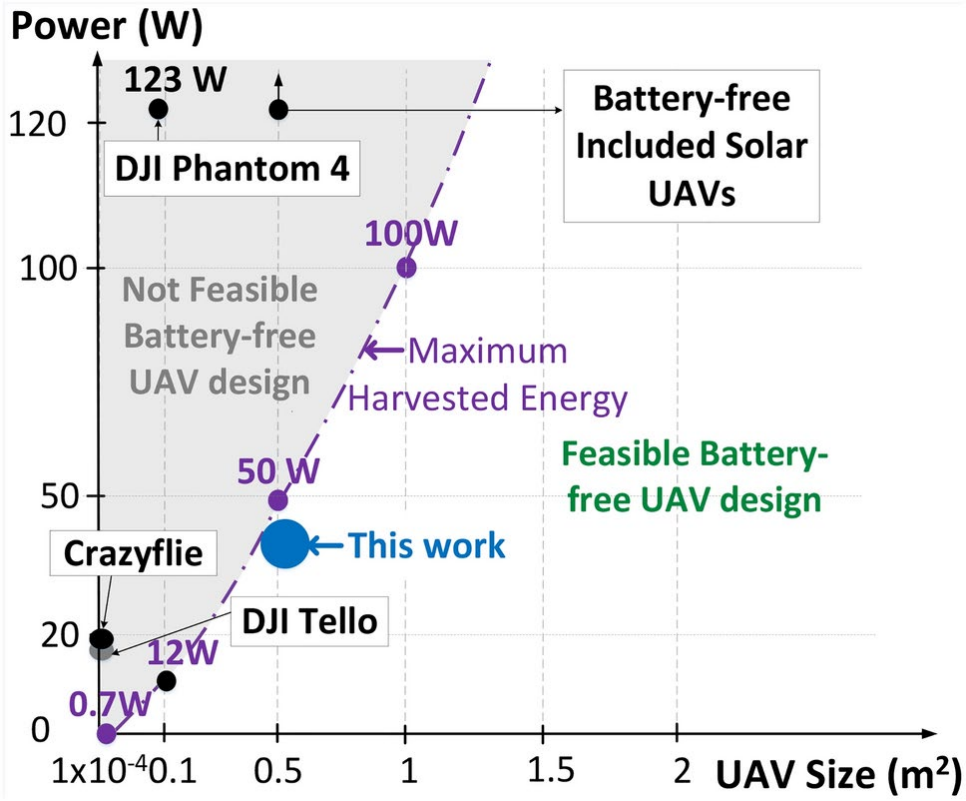
An analysis on COTS solar harvesting efficiency and battery-free UAV surface area.

*Feasibility analysis* Building a battery-free UAV with COTS materials and components comes down to balancing power consumption, lift force generation, and weight. The power required to sustain flight in a fixed-wing UAV can be minimized through efficient aerodynamic design and lightweight materials. Fixed-wing UAVs generate lift through their wings, which allows them to glide and maintain control even with intermittent power. By using lightweight COTS materials, the overall weight of the UAV can be kept low, reducing the power needed to maintain lift. Advances in solar panel efficiency and lightweight construction materials make it possible to harvest and store sufficient energy to power the UAV’s systems. Figure 14 describes the relationship of the energy that can be produced by different sizes of UAVs (DJI Phantom^81^, DJI Tello^82^, Crazyflies^83^, and others^43^) using available COTS solar cells. Note that this analysis does not account for the imperfections in solar cells, materials, fabrication tools, and the aerodynamic behavior of UAVs. Consequently, while the development of battery-free UAVs is feasible, there are numerous challenges that must be addressed to validate the viability of this concept.

### Proposed approach

*Design principle and challenges* The goal of this work is to create a robust battery-free fixed-wing UAV to perform a full cycle take-off, fly, and landing safely just by using solar energy. The design considerations focus on achieving sustainable and efficient flight through novel energy harvesting and management techniques, as well as lightweight construction. The UAV must be able to harvest, store, and utilize solar energy without relying on lithium batteries. Additionally, it should achieve stable-level flight using its own power. An important capability of the drone is to detect intermittent solar events and adjust its flight planning accordingly to ensure continuous operation. These objectives collectively aim to develop a practical and sustainable battery-free UAV.

*Challenges* However, realizing this UAV presents several technical challenges that would need to be addressed:

- Buffering solar energy without batteries is difficult. Typically, any power produced by a solar array can be pushed directly into the charge controller of a battery; however, batteries are not allowed and the technology to efficiently charge capacitors from solar panels will need adaptation.
- Modifying every UAV airframe for a battery-free approach will not be feasible. Multirotor UAVs can have high power requirements and low real-estate for solar panel deployment. Selecting the right airframe will be key.
- Performing sensing, computing, communication, and actuating with intermittent power are extremely challenging. Solar panels operate most effectively when they receive solar radiation directly normal to their surface. Because any UAV will need to maneuver in several orientations during nominal flight, techniques to mitigate power failures due to poor maneuvering choices and other intermittent events must be developed.

**Fig. 15:**
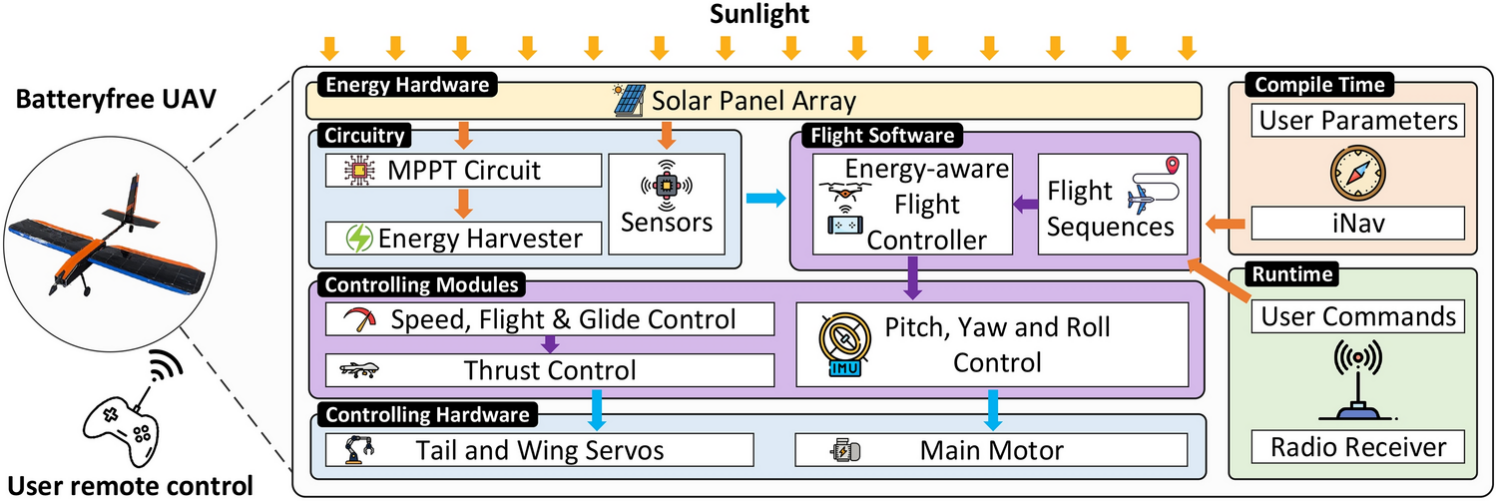
System overview.

**Fig. 16:**
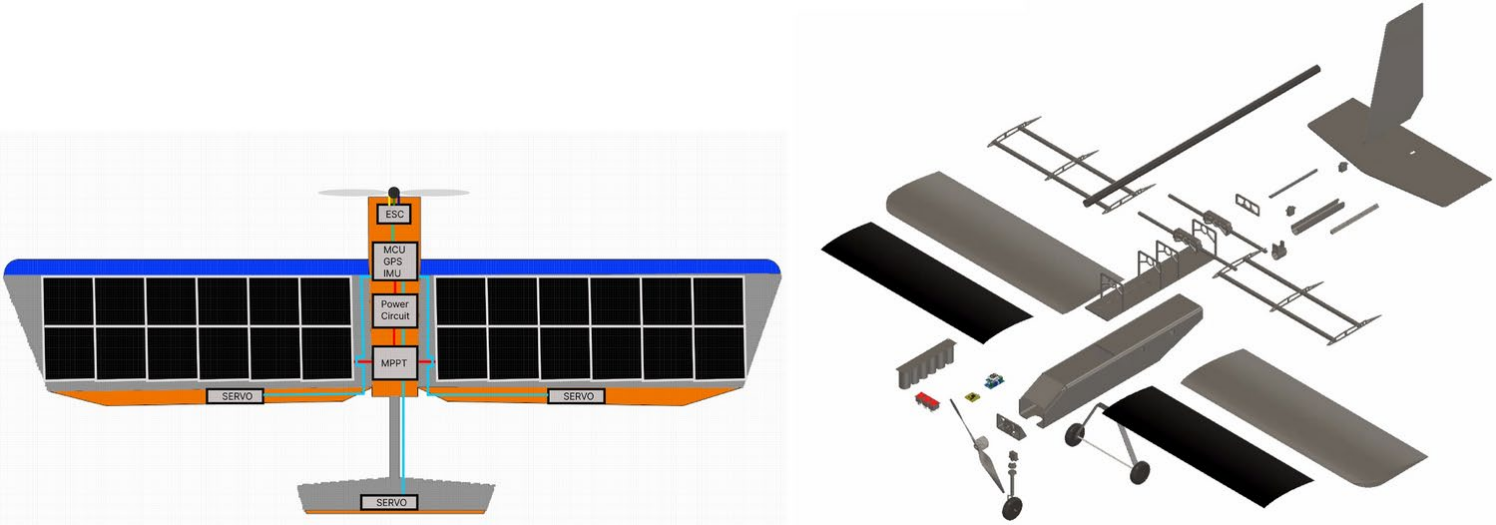
Schematic (left) and 3D Design (right) of the proposed battery-free UAV. The schematic was created using the Figma drawing tool^84^, and the 3D design was developed using Autodesk Fusion 360^85^, both by the authors under educational licenses.

**Fig. 17:**
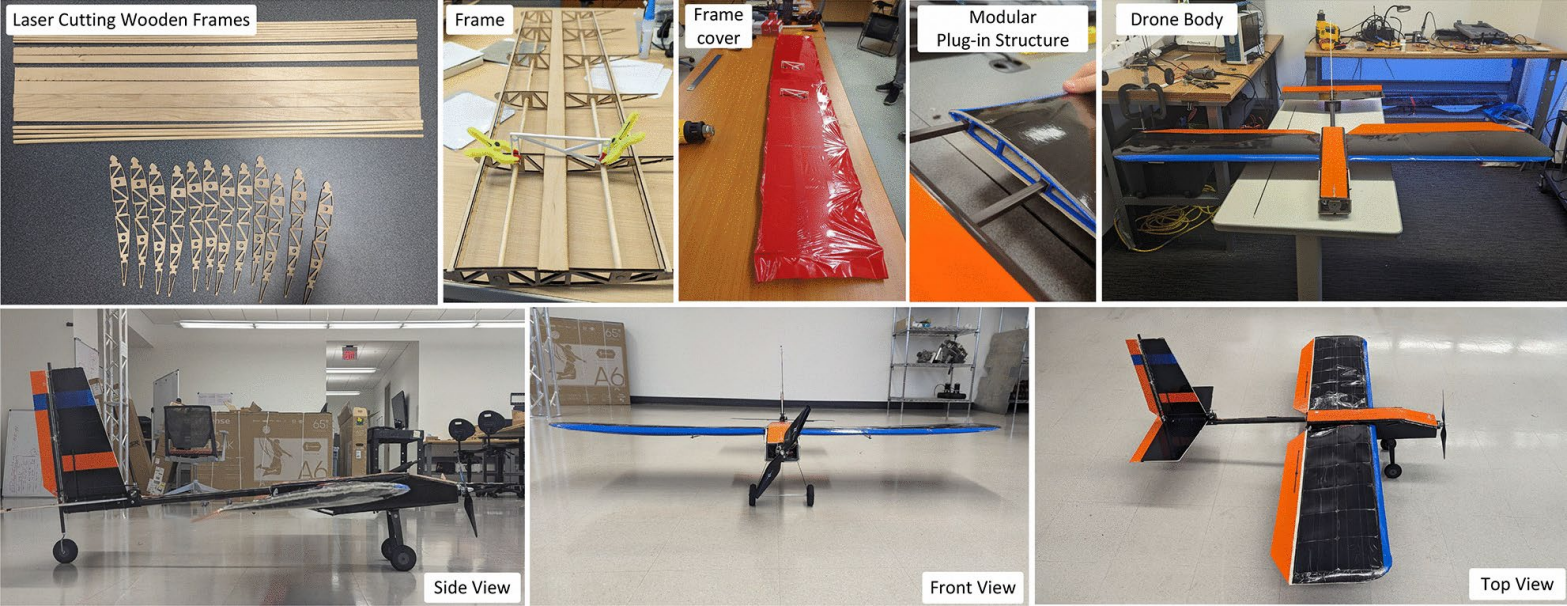
Process of fabricating the batteryless UAV prototype using COTS components.

### System overview

The proposed battery-free UAV architecture is illustrated in Fig. 15, with a physical schematic provided in Fig. 16. This architecture integrates three key components: the airframe, power system, and control algorithms. These components are interconnected, with each imposing specific design constraints that influence the overall UAV performance and functionality. The airframe is optimized for lightweight durability and aerodynamic efficiency, the power system is tailored to manage energy harvesting and distribution effectively, and the control algorithms ensure stable flight under dynamic environmental conditions. Together, these elements form a cohesive system designed to overcome the challenges of battery-free operation while meeting the mission goals of sustained and reliable UAV performance.

#### Airframe

*3D modeling, design, and fabrication* We design, fabricate, and implement techniques for robust fixed-wing battery-free UAV leveraging commercial-off-the-shelf (COTS) materials and rapid prototyping manufacturing techniques. All the designs are calculated, simulated and generated from computer-Aided Design (CAD). The airframe provides structure and strata for thrust and lift generation. The main components are the fuselage, the wings, and the tail. All of these components are fabricated from laser-cut vinyl-reinforced polystyrene sheets which are formed to shape as necessary. The fuselage is constructed with an internal compartment which houses electrical and control components. This compartment is reinforced with 3D-printed forms which add rigidity and attachment structures for the tail and wings. Figure 17 illustrates the process of fabricating the UAV and its final prototype. The tail of the UAV uses a flat-plate convention for the horizontal and vertical stabilizers, both of which are affixed to a 3D-printed form which fits over a carbon-fiber reinforced-polymer (CFRP) tube. This tube acts as a tail boom, holding the tail aft of the fuselage with minimal structure and weight. The UAV is designed with a conventional fixed-wing aircraft flight control system. A vertical rudder is hinged aft to the vertical tail stabilizer, a horizontal elevator is hinged aft to the horizontal tail stabilizer, and an aileron is hinged aft to each of the wings. These control surfaces lend pitch, roll, and yaw control to the aircraft’s flight control algorithms.

*Thrust development* The powerhouse of the prototype is the Mn3110-17 Tiger Motor. Table 3 gives a factory accounting of the power requirements and outputs of the motor given a 13x4.4 dual-bladed propeller. Given the 86.4 W available, a comfortable 80% of the motor’s power would be available giving a conservative 750 grams-force of thrust.

*Wing development* The MH117 airfoil was chosen as the basis of the wing for a combination of high lift-to-drag ratio and simple geometry conducive to solar array mounting. Tabulated performance data for the MH117 airfoil is readily available^86,87^. Given an estimated maximum thrust a theoretical maximum takeoff weight can be derived. Although the UAV is capable of producing more than 750 grams-force of thrust, this metric is chosen because it is predicted to be the maximum thrust the UAV is capable of producing indefinitely in favorable solar conditions based on the output of the solar array and the data in Table 3. Note that parasitic drag sources due to body elements like landing gear are not examined in this derivation. Without a doubt, these drag sources are not negligible for the prototype developed, but these types of drag cannot be well characterized without highly specialized testing equipment.

The following derivation is used to predict flight characteristics for the proposed UAV in steady level flight^88^:

The maximum velocity will occur when the drag forces equal the available thrust. Given our estimated maximum thrust of $T$ = 750 grams-force, we can estimate the maximum velocity of the UAV by substituting the equation for drag force:

$$\begin{aligned} T = D = \frac{1}{2} \rho v_\text {max}^2 S C_D \end{aligned}$$

where $\rho$ is the air density at sea level, typically $\rho = 1.225 \, \text {kg/m}^3$, $v$ is the velocity of the airflow over the wing, measured in $\text {m/s}$, $S$ is the wing area (for our wing scenario, $S = 0.4368 \, \text {m}^2$), $C_L$ is the coefficient of lift (for our wing scenario, $C_L = 1.2$), $C_D$ is the coefficient of drag. For our wing scenario, $C_D = 0.03$. Solving this equation for $v_\text {max}$ we get:

$$\begin{aligned} v_{\text {max}} = \sqrt{\frac{2T}{\rho S C_D}} \approx 30.26 \ m/s \end{aligned}$$

With an estimated $v_\text {max}$, we can estimate the maximum takeoff weight for the UAV. The maximum takeoff weight will be equal to the lift at $v_\text {max}$:

$$\begin{aligned} W = L = \frac{1}{2} \rho v^2 S C_L \approx 29700 \ \text {grams-force} \end{aligned}$$

**Fig. 18:**
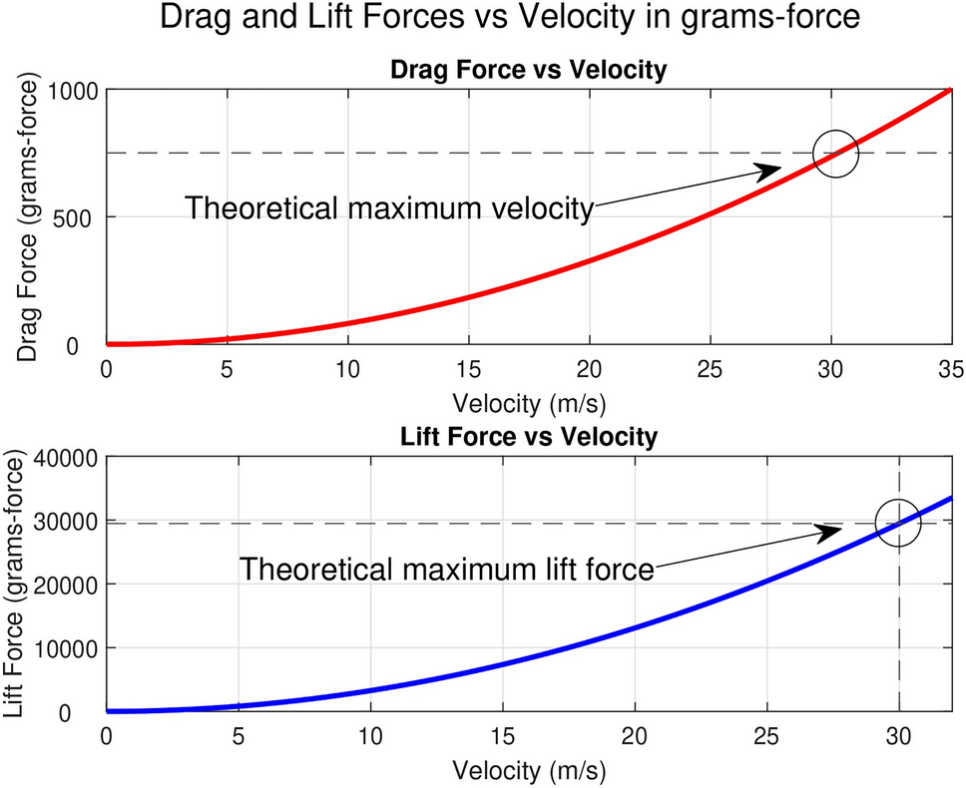
Graphical derivation of maximum velocity and lift force.

Figure 18 summarizes this approach graphically, showing a theoretical top speed of 30.26 m/s and theoretical max takeoff weight of 29.7 kg. These predictions are based on the assumptions that a constant 750 grams-force is available and that parasitic drag sources are negligible.

**Table 3:** Mn3110-17 motor performance at 11.1 V^89^.

Throttle	Watts (W)	Thrust (g)	RPM	Efficacy (g/W)
50%	25	320	3500	12.80
65%	47	480	4500	10.21
75%	69	680	5000	9.86
85%	92	880	5600	9.57
100%	111	980	6000	8.83

#### Power management system

**Fig. 19:**
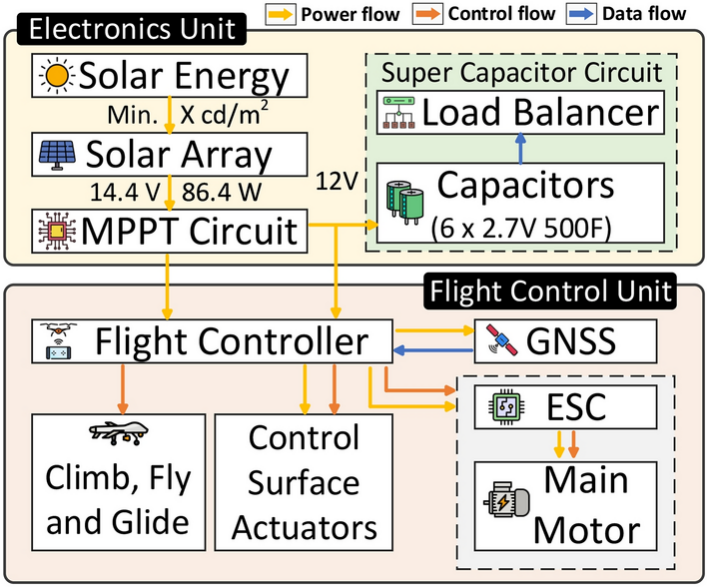
Energy management unit.

Figure 19 summarizes the the energy flow for the UAV. The UAV presented is equipped with a custom 86.4 W solar array comprised of 24 Sun Power C60 mono-crystalline cells. Solar cells exhibit constant current behavior through the majority of their useful voltage curve, however a sharp reduction in current occurs at the upper limit of the cell’s voltage capability. The maximum power transfer point occurs and can be identified continuously using control circuits. To this end, a MAX20801CEVKIT maximum power point tracking module is utilized. This module continuously extracts the maximum power attainable from the solar array and outputs 12V while preventing current from back-flowing into the solar array. Because solar energy is intermittent, super-capacitors are utilized to store excess energy until it is needed. The super-capacitor bank used is a series array of 6 2.7V 500F capacitors with a typical equivalent series resistance (ESR) of 5 mOhms. The 12V output of the MPPT module is applied over the array of capacitors, and a balancing circuit leaks charge between the individual capacitors to ensure that manufacturing defects in ESR do not cause charge imbalances in the bank. Due to variations in capacitance, ESR and connections the capacitors connected in series can be charged unevenly. This could lead to a situation where one of the capacitors reaches its maximum voltage while others can still be charged. This can lead to capacitors getting overcharged and losing power. To prevent this issue we connected a MOSFET parallel to each capacitor to implement a FET Balancing circuit as implemented in the ALD810026SCL chips. Upon reaching a threshold voltage difference between 2 capacitors connected in series, the MOSFET is turned on allowing the extra current to flow through the MOSFET slowing down the charge of the overvolting cell. This is done for every cell in the stack to ensure the voltage of each cell in the stack is within a reasonable amounts. Further, because the series voltage of the bank is 16.2V nominal and the MPPT module only produces 12V, there is no danger of over-volting the capacitors. The capacitor bank is installed in a buffer configuration, supplying the flight controller with extra power during peak usage.

#### Control systems

The control system used in the UAV is a Matek F405-WMN flight controller. This flight controller is a popular commercial solution for hobby RC aircraft and is designed with I2C, five UART channels, 12 pulse-width modulation (PWM) generators, dual-dedicated video transmission outputs, three on-board ADCs, a 16MB flash storage, and an entire community of solutions ready to integrate into the open-source programming interface iNav. iNav enables a GUI-based approach to configuring several typical UAV airframes, including the conventional fixed-wing used in this work. Additionally, iNav includes a logical programming interface enabling user-defined algorithms to monitor flight and power conditions and intervene when necessary. The flight controller is capable of performing flight autonomously, but for testing and prototyping a radio control (RC) link was utilized. A modest 10-channel RC transmitter and receiver pair enabled full motor and control surface functionality alongside several programmatic control switches that could be operated from the handheld controller. The UAV was programmed with a conventional fixed-wing configuration in iNav, assigning control of each of the four control surface servos and the thrust motor to specific RC channels. A safety arming switch required the use of one additional RC channel, leaving four RC switches to be used in algorithmic control and navigation modes. For the testing in Section 7, a single RC switch was used to engage energy-aware control algorithms.

*Energy-aware control algorithms* Traditional flight controllers do not consider energy available in the system as a constraint. This is not the case when we are working with intermittent flight and we need a way to track and adapt to the energy available in the system. To address this issue we developed and evaluated two different energy aware algorithms to supplement existing flight controllers. These current algorithms only change the amount of throttle to control energy expenditure. It is worth noting that if the energy in the system becomes too low the throttle of the UAV will be shut off completely; however, this is not necessarily a critical state since the system will still be capable of managing flight control surfaces for minutes or more after the main motor is off, affecting stable glides from which recovery is possible once intermittent periods are over.

*Greedy energy-aware control* (GEAC) (Algorithm 1) sets a high and low threshold to the capacitor voltage. If the capacitor voltage drops below the low threshold the throttle is decreased and if the capacitor voltage rises above the high threshold throttle can be increased. Throttle can be altered while in the range of 0 to max throttle where max throttle can be set by the pilot in control. This algorithm helps in preventing brown outs by limiting the energy output to the motors when the system becomes close to undervolting, however it frequently experiences total loss of thrust events as a result of power conversation. It is preferable to keep the motor spinning, even with very low thrust, over longer periods of time rather than engaging the motor many times in the same period because the inrush current of the motor at a stop is highly inefficient. To this end, a further algorithm was developed.

*Predictive energy-aware control* (PEAC) (Algorithm 2) tracks the capacitor voltage as well as how the voltage has been changing, i.e., are the capacitors charging or discharging. The algorithm extends on algorithm 1 by slowly decreasing throttle as the capacitor voltage reaches the low threshold and slowly increasing throttle as the capacitor voltage approaches the high threshold. This helps to prevent total-loss-of-thrust events when harvested energy is unstable.

**Algorithm 1:**
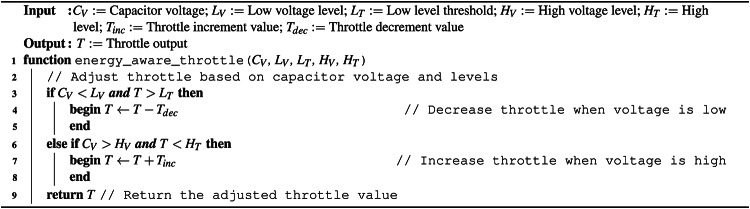
Greedy energy-aware algorithm.

**Algorithm 2:**
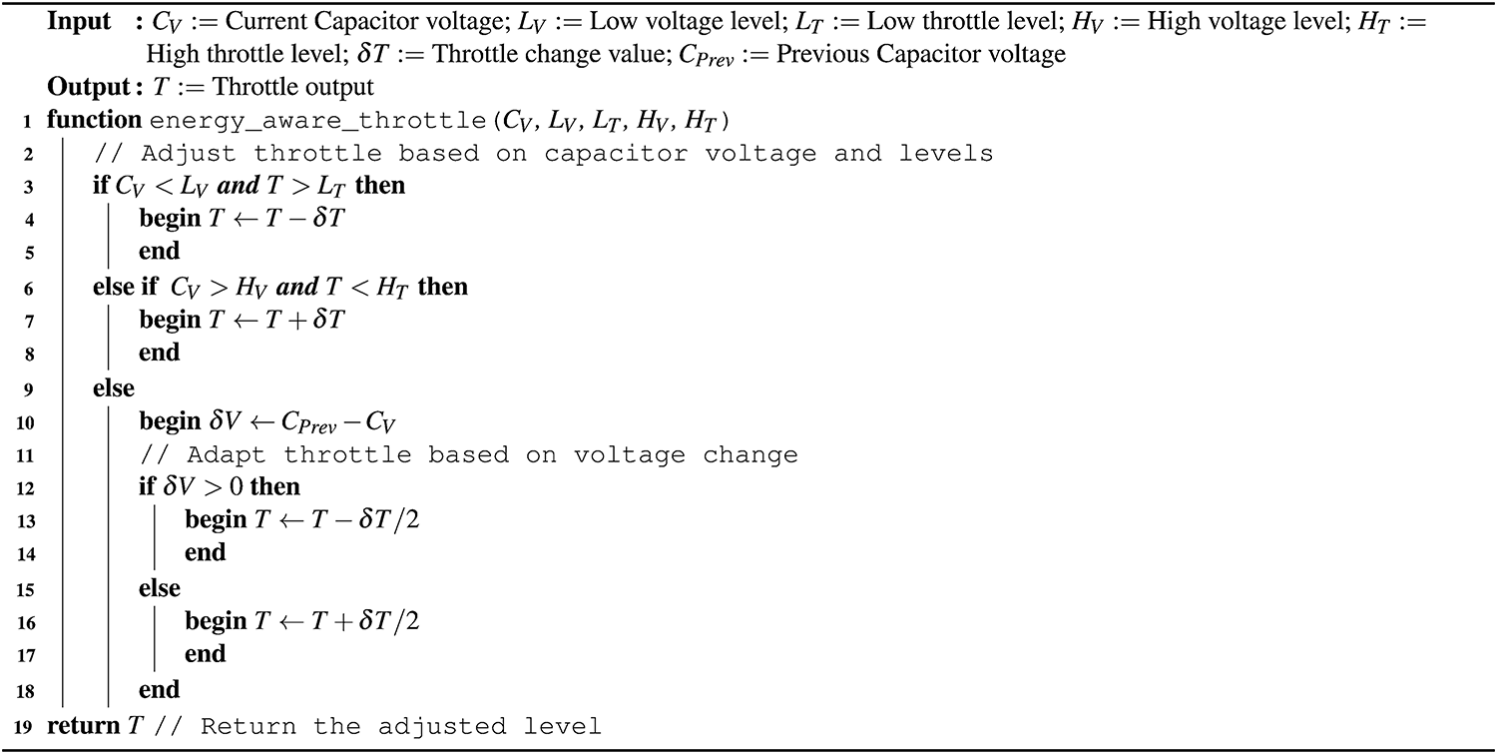
Predictive energy-aware algorithm.

### Validation

**Fig. 20:**
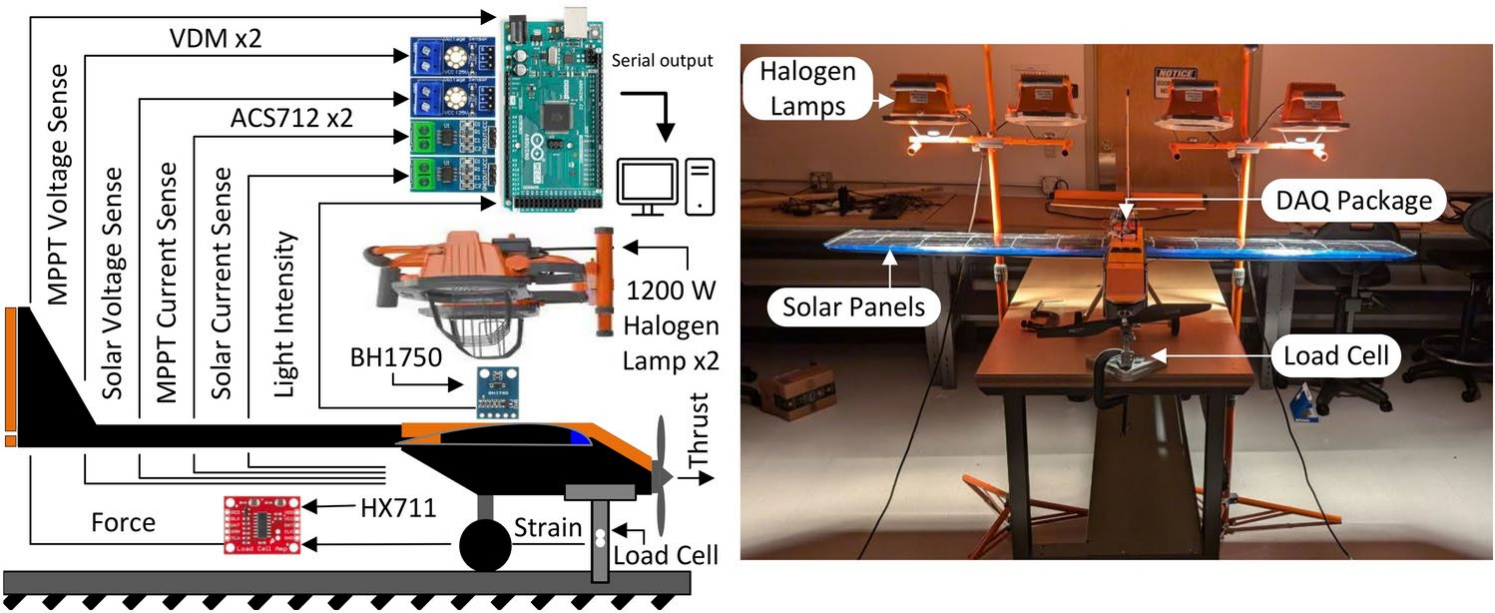
Experimental setup and data acquisition package for lab setting experiments.

The batteryless UAV design was validated in a lab setting (Fig. 20) and an outdoor setting. In the lab setting, a data acquisition platform was developed using an Arduino Mega MCU and COTS sensors with open-source integration solutions for the Arduino platform. The data acquisition package was equipped with two analog voltage detection modules (VDMs) which used precision resistors in a voltage divider configuration to read the voltage of the solar array and the voltage of the MPPT output with a 0.00489 V resolution. The data acquisition package also used two ACS712 modules to measure the corresponding currents from the solar array and MPPT output. The batteryless UAV was mounted in a roller-cantilever configuration with a 10 kg load cell whose strain was measured and converted by an HX711 load cell amplifier to observe the thrust developed by the UAV. Finally, a BH1750 light intensity module was integrated to measure the simulated solar radiation. To simulate solar radiation, a pair of 1200 W halogen work lights were positioned above the wings at a distance of approximately 30 cm, a happy compromise between radiation density and heat management concerns. All data that refers to in lab experiments was performed using this data acquisition package in a closed lab room with minimal ambient light fluctuations.

To perform outdoor experiments, the batteryless UAV was disassembled and transported to a local stadium parking lot which was suitably clear of foliage and vehicles. After reassembly, the UAV was operated using the handheld radio transmitter. A handheld light intensity meter was used to record solar intensity and flights were attempted with video recordings both onboard and standby.

## Conclusion

This work demonstrates the feasibility of a battery-free solar-harvesting UAV. We designed and fabricated a fixed-wing UAV powered entirely by solar energy, managed by novel energy-aware control algorithms. A thorough analysis proves the system can harvest enough energy in real-time for flight. We also show the efficacy of GEAC and PEAC in mitigating power brownouts and total loss of thrust events. In favorable solar conditions, the UAV can produce 350 grams-force of continuous thrust indefinitely and achieve a speed of 15 m/s under its own power.

While we are excited about the current progress, further research will be conducted to increase the robustness of battery-free UAVs. We now highlight a few immediate action research items. First, although the solar array was specified to generate up to 86.4 W of power, the experiments performed could only generate half of this metric even in outdoor testing. Second, the voltage level of the super capacitors clamps the power available to the flight controller, lowering overall thrust output. This phenomenon can be mitigated with control algorithms, but a circuit solution would be preferable. Third, while the airframe was capable of handling the stresses of flight, there are many sources of parasitic drag that can be minimized to more closely reconcile the lift and drag analysis. For example, the fuselage is not designed with any airfoil shape and can be a severe source of parasitic drag. Lastly, while the UAV is only suitable for flying at certain hours and weather conditions, this is an important milestone, and extending its robustness is our next research step.

## Data Availability

The datasets used and/or analyzed during the current study available from the corresponding author on reasonable request.
